# Supplementary material for: Iron Bioavailability in the Extracellular Environment Is More Relevant Than the Intracellular One in Viability and Gene Expression: A Lesson from Oligodendroglioma Cells
Source: Biomedicines. 2023 Oct 31;11(11):2940. doi: 10.3390/biomedicines11112940 (PMC10668974; doi:10.3390/biomedicines11112940)
Supplement: Supplementary file 1 [file biomedicines-11-02940-s001.zip › biomedicines-2636917-supplementary.pdf]

Supplementary Material and Methods

Confocal Laser Scanning Microscope analysis (by ALEMBIC, IRCCS San Raffaele and Università Vita-Salute San Raffaele)

Cells were seeded onto 25 mm diameter glass-coverslips (CLS1760-025 Chemglass Life Sciences LLC, USA) in complete medium supplemented or not supplemented with ferric citrate.
Before the analysis, cells were rinsed twice in PBS and incubated for one hour in a complete medium supplemented or not with 10 μM of ferric citrate in the presence of 5 μM Rhonox-1. For nuclei counterstaining, 5 μg/mL of Hoechst 33-258 was added to all the tested conditions during the last 20 minutes of incubation.
All images were collected with an Olympus fluoVIEW FV3000RS Confocal microscope equipped with a UPLSAPO 60XS (NA 1.3) silicone oil lens single confocal sections adopted independent configuration settings to avoid possible crosstalk during acquisition. Fe^{2+} and Hoechst 33-258 were excited at 405nm LL (laser line) with a spectral detection ranging from 430nm to 470nm. The emission of Rhonox-1 signal excited at 561 nm LL was collected with a spectral detection ranging from 570 to 620nm.

Supplementary Results

Effect of medium composition on cell models

	**Fe fg/cell (mean±SD)**	
	**HOG**	**HOG 100 μM**
LG-DMEM-1%FBS-0 μM ferric citrate	31.72±7.59	1776.91±576.17
LG-DMEM-1%FBS-25 μM ferric citrate	122.51±29.86	1816.93±386.79
LG-DMEM-1%FBS-100 μM ferric citrate	597.6±83.8	2439.8±722.3

Table S1. Effects of 24-hour incubation of medium with different iron concentrations on the intracellular iron content of HOG cells.

Confocal microscopy confirms the iron uptake

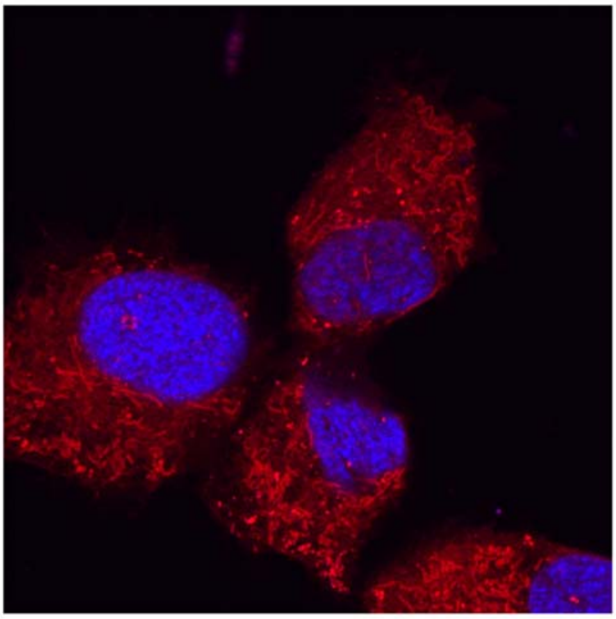

Figure S1. Confocal images of HOG100 cells in 10 μM of ferric citrate**.** Cells were cultured in a standard culture medium in the presence or absence of ferric citrate 10 μM for 24 hours. Thereafter, cells were incubated for one hour in a complete medium supplemented or not with 10 μM of ferric citrate in the presence of 5 μM Rhonox-1 (red). For nuclei counter-staining, 5 μg/mL of Hoechst 33-258 (blue) was added to all the tested conditions during the last 20 minutes of incubation. Rhonox-1 signal, linked to Fe^{2+}, is mainly compartmentalized. Images were collected with an Olympus fluoVIEW FV3000RS Confocal microscope.
